# Chemoreflex Control as the Cornerstone in Immersion Water Sports: Possible Role on Breath-Hold

**DOI:** 10.3389/fphys.2022.894921

**Published:** 2022-06-06

**Authors:** Alexis Arce-Álvarez, Camila Salazar-Ardiles, Carlos Cornejo, Valeria Paez, Manuel Vásquez-Muñoz, Katherine Stillner-Vilches, Catherine R. Jara, Rodrigo Ramirez-Campillo, Mikel Izquierdo, David C. Andrade

**Affiliations:** ^1^ Exercise Applied Physiology Laboratory, Centro de Investigación en Fisiología y Medicina de Altura, Departamento Biomedico, Facultad de Ciencias de La Salud, Universidad de Antofagasta, Antofagasta, Chile; ^2^ Escuela de Kinesiología, Facultad de Salud, Universidad Católica Silva Henríquez, Santiago, Chile; ^3^ Navarrabiomed, Hospital Universitario de Navarra (CHN), Universidad Pública de Navarra (UPNA), IdiSNA, Pamplona, Spain; ^4^ Clínica Santa María, Santiago, Chile; ^5^ Exercise and Rehabilitation Sciences Laboratory, School of Physical Therapy, Faculty of Rehabilitation Sciences, Universidad Andres Bello, Santiago, Chile

**Keywords:** water sports, peripheral chemoreflex, central chemoreflex, autonomic nervous system, apnea

## Abstract

Immersion water sports involve long-term apneas; therefore, athletes must physiologically adapt to maintain muscle oxygenation, despite not performing pulmonary ventilation. Breath-holding (i.e., apnea) is common in water sports, and it involves a decrease and increases PaO_2_ and PaCO_2_, respectively, as the primary signals that trigger the end of apnea. The principal physiological O_2_ sensors are the carotid bodies, which are able to detect arterial gases and metabolic alterations before reaching the brain, which aids in adjusting the cardiorespiratory system. Moreover, the principal H^+^/CO_2_ sensor is the retrotrapezoid nucleus, which is located at the brainstem level; this mechanism contributes to detecting respiratory and metabolic acidosis. Although these sensors have been characterized in pathophysiological states, current evidence shows a possible role for these mechanisms as physiological sensors during voluntary apnea. Divers and swimmer athletes have been found to displayed longer apnea times than land sports athletes, as well as decreased peripheral O_2_ and central CO_2_ chemoreflex control. However, although chemosensitivity at rest could be decreased, we recently found marked sympathoexcitation during maximum voluntary apnea in young swimmers, which could activate the spleen (which is a reservoir organ for oxygenated blood). Therefore, it is possible that the chemoreflex, autonomic function, and storage/delivery oxygen organ(s) are linked to apnea in immersion water sports. In this review, we summarized the available evidence related to chemoreflex control in immersion water sports. Subsequently, we propose a possible physiological mechanistic model that could contribute to providing new avenues for understanding the respiratory physiology of water sports.

## Introduction

Immersion sports, such as apnea diving, artistic swimming, classical swimming, or long-term swimming, involve acute and chronic cardiorespiratory and muscular adjustments (Viana et al., 2019; Wasfy et al., 2019; Elia et al., 2021). All of these sports activities involve continuous and/or intermittent long-term apneas that are concomitant with stern exercise efforts during several training sessions and competitions (Guimard et al., 2014). Of note, and in contrast to land sports, swimmer athletes are able to maintain O_2_ supplies to active tissues during exercise, although they do not perform pulmonary ventilation for several seconds or minutes (approximately 4 min) (Heusser et al., 2009), which could confer robust storage/delivery oxygen to active muscles (Schagatay et al., 2000; Andersson et al., 2002; Engan et al., 2013; Konstantinidou and Chairopoulou, 2017). Similarly, it has been demonstrated that during a maximum breath-hold, alveolar O_2_ ventilation can decrease to 30 mmHg, and oxygen saturation can also decrease to 50% (Ferretti et al., 1991). However, despite the abrupt decrease in alveolar ventilation and arterial desaturation, skeletal muscle functionality is preserved (Ferretti, 2001; Kjeld et al., 2018). In addition, arterial CO_2_ accumulation and the decrease in O_2_ during a breath hold can stimulate central and peripheral chemoreceptors, respectively, thus triggering autonomic and cardiovascular adjustments, which may correspondingly uncouple the end of apnea (Dempsey et al., 2012; Kumar and Prabhakar, 2012).

The principal peripheral chemoreceptors are the carotid bodies (CB), which are bilaterally located on the common carotid artery bifurcation (Iturriaga et al., 2021). This organ is composed of chemoreceptor type I cells and glial cells (type II cells) (Iturriaga and Alcayaga, 2004; Iturriaga et al., 2021). Carotid body type I cells are considered to be polymodal receptors and can respond to several stimuli, due to the fact that it is the main component of the homeostatic acute oxygen-sensing system required to trigger cardiorespiratory and ventilatory adjustments during hypoxemia (López-Barneo et al., 2008; Iturriaga et al., 2016; López-Barneo et al., 2016). Furthermore, central chemoreceptors are found in different areas of the brainstem; nevertheless, it has been proposed that the retrotrapezoid nucleus (RTN) is the more important site for regulating central chemosensitivity to CO_2_/H^+^ (Guyenet, 2012). Central respiratory chemosensitivity refers to the homeostatic reflex by which brainstem circuits regulate breathing in response to changes in CO_2_ or its proxy H^+^ (Kumar et al., 2015). Although peripheral/central chemoreceptors could be determinants of the maximum apnea duration, there is no conclusive evidence showing their role in apnea duration in water sports. Nevertheless, we recently showed that, in accordance with a longer apnea duration, the chemoreflex is reduced in swimmers’ athletes compared to a control condition (Arce-Álvarez et al., 2021). Of note, this adaptative process is not only observed in mammals, if not also in amphibian and reptile species (Santin, 2017), showing neuroplasticity impacting CO2/O2 chemoreflex. Indeed, it has been speculated to increase breath-hold duration to lengthen dive time adaptively for these animals. Therefore, and considering this parallelism between mammals and amphibians, it is possible to propose that peripheral/central chemoreflex function partially governs the apnea duration in swimmer and/or diver athletes. Therefore, and considering this parallelism between mammals and amphibians, it is possible to propose that peripheral/central chemoreflex function partially governs the apnea duration in swimmer and/or diver athletes. Nevertheless, although several mechanisms have been proposed to explain apnea and its breakpoint, this mini-review focuses on the responses and possible adaptations of the central and peripheral chemoreflex and their possible role to activate the spleen in maintaining respiration; additionally, we will explore and discuss potential mechanisms to explain a breath-hold in divers and swimmers’ athletes.

## Peripheral and Central Chemoreflex Control

### Peripheral Chemoreflex

**FIGURE 1:**
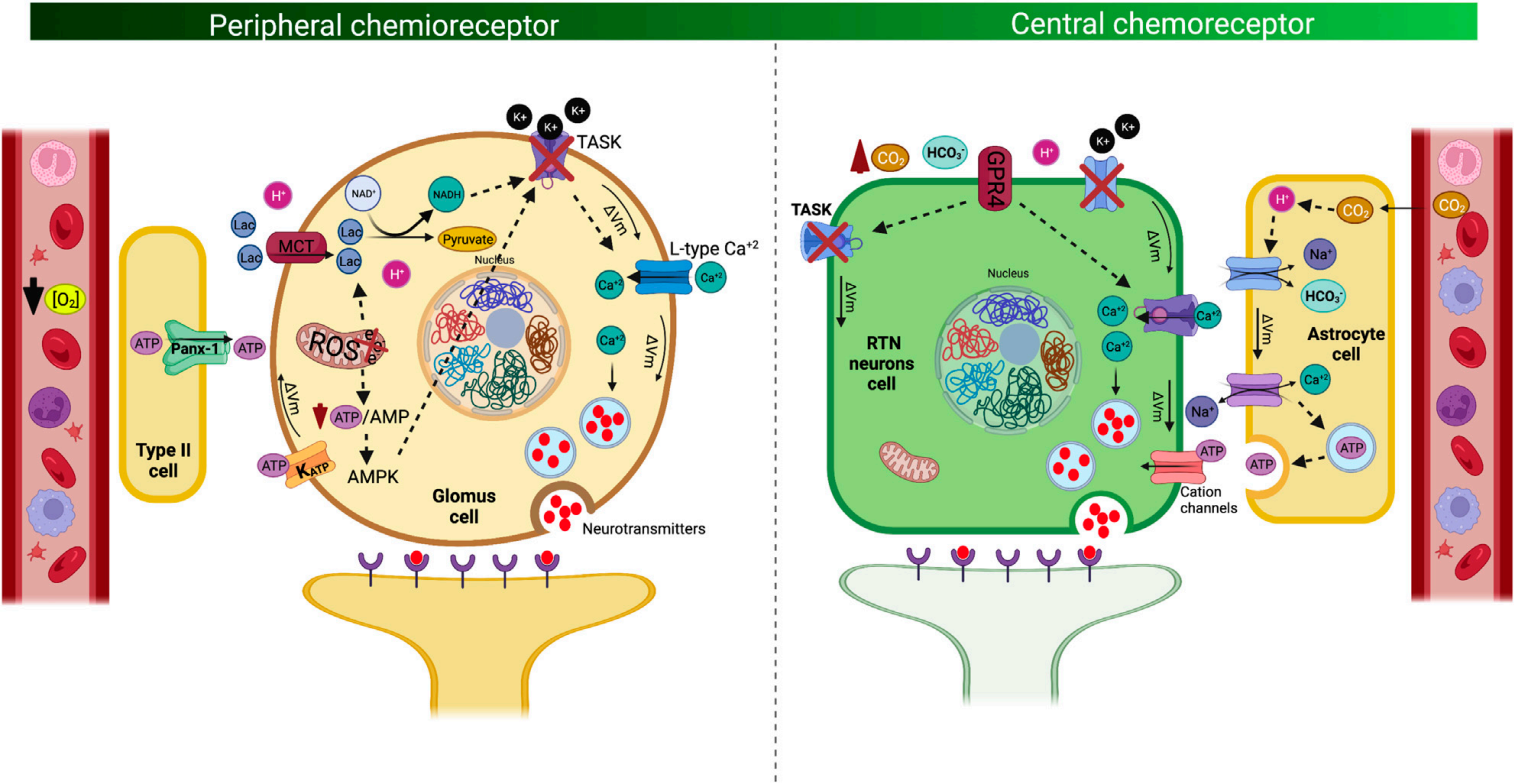
Mechanism of signal transduction and cell excitability of the peripheral chemoreceptor cells. Peripheral chemoreflex signal transduction mechanism in which type 1 glomus cells are activated by hypoxia (i.e., decrease O_2_ bioavailability) and metabolic stress (i.e., lactate) (left panel). Of note, hypoxia inhibits the mitochondrial electron transport, decreasing ATP production, which promotes lactate production and activation of adenosine monophosphate kinase (AMPK). Metabolic shift-dependent lactate production and accumulation which is transported through monocarboxylate cotransporter type 1 (MCT1) increase NADH production, concomitant to AMPK, are the molecular entities responsible for closing K^+^ channels, activating voltage-dependent calcium channel (Ca^+2^ influx to the cell). In addition, type II cells express Pannexin 1 (Panx-1), by which ATP is released, affecting ATP-dependent K^+^ channels. All this mechanism contributed to induces the release of neurotransmitters such as acetylcholine, dopamine, and adenosine by exocytosis to the carotid sinus nerve. Mechanism of signal transduction and cell excitability of the Central chemoreceptor cells (right panel). Neurons from the retrotrapezoid nucleus (RTN) are activated by CO_2_ and its proxy H^+^ in the cerebrospinal fluid (pH-sensitive). Acidosis activates the related G protein-coupled receptors 4 (GRP4), which produces closure of tandem pore domain in weakly rectifying K^+^ channels (TWIK), consequently depolarizing the membrane and opening of Ca^+2^ channels, inducing the release of neurotransmitters by exocytosis, promoting hyperventilation and sympathoexcitation. In addition, RTN neurons possibly also respond to changes in extracellular HCO_3_^−^ concentration by a K^+^-independent mechanism. Of note, central chemotransduction, algo could be dependent on the astrocytes. The increase of PaCO2, promotes an increase of H^+^ production activating the Na^+^/Ca^2+^ exchanger (NCX), producing the influx of Ca^2+^, which allows the release of ATP by exocytosis from the astrocyte towards extracellular space. The ATP released by the astrocytes activates purinergic receptors of the RTN chemoreceptor neurons, triggering depolarization of these neurons and stimulating the central pattern generator (CPG).

The carotid body is a bilateral sensory organ, with an estimated average volume of approximately 20 mm^3^, and it is located in the common carotid artery bifurcation and innervated by the carotid sinus nerve (Iturriaga et al., 2021; Forbes and Menezes, 2021; Joyner et al., 2018; Ortega-Sáenz and López-Barneo, 2020). These organs are the main oxygen sensors and mostly respond to hypoxemia, thus promoting hyperventilation and sympathoexcitation, which contribute to restoring arterial blood gas homeostasis (Iturriaga et al., 2016; Iturriaga et al., 2021; Ortega-Sáenz and López-Barneo, 2020). CB chemoreceptors are composed of chemoreceptor type I cells and glial cells (type II cells) (Iturriaga and Alcayaga, 2004; Iturriaga and Alcayaga, 2021; Ortega-Sáenz and López-Barneo, 2020). CB type I cells are considered to be polymodal receptors that respond to hypoxemia, hypercapnia, acidosis, blood flow, temperature, leptin and insulin concentrations, osmolality, and lactate; additionally, they are the main component of the homeostatic acute oxygen-sensing system required to produce cardiorespiratory and ventilatory adjustments during several stimuli (López-Barneo et al., 2008; Iturriaga et al., 2016; Iturriaga et al., 2021; Ortega-Sáenz and López-Barneo, 2020; Torres-Torrelo et al., 2021). Furthermore, type II cells or glia-like stem cells mainly have a functional role, and although their function is not completely known, they have been associated with the adaptive growth processes of CB in prolonged hypoxic stimuli (Xu et al., 2003; Pardal et al., 2007). Several hypotheses have been proposed to explain the mechanism related to the excitation of CB type I cells during hypoxia (Rakoczy and Wyatt, 2018). Chang et al. (2015) proposed that hypoxia inhibits electron transport of the mitochondria, which favors the formation of lactate, which activates the Olfr78 receptor, producing the inhibition of K^+^ channels, depolarizing the cell, and releasing neurotransmitters in CB type 1 cells (Chang et al., 2015). In addition, Wyatt and Buckler (2004), propose that hypoxia-induced inhibition of electron transport reduces ATP production and closes weakly rectifying K^+^ channels (TWIK)-related acid-sensing K^+^ (TASK), promoting depolarization of the cell and the release of neurotransmitters (Wyatt and Buckler, 2004). Interestingly, it has been proposed that the inhibition of mitochondrial electron transport and the consequent fall in ATP activates AMPK, which phosphorylates membrane ion channels causing depolarization and release of neurotransmitters (Evans et al., 2016; Wyatt et al., 2007). On the other hand, it has been proposed that hypoxia is able to inhibit mitochondrial electron transport, which increases reactive oxygen species, reducing the nucleotides of mitochondrial complex 1, modifying the redox state of the membrane channels, exciting the cell (Fernandez-Aguera et al., 2015). However, it is important to mention that independent of all these possible mechanisms, The most accepted mechanism related to glomus cell depolarization during hypoxia (PaO2 below 60 Torr or pH below 7.20) is related to decreased K+ permeability and increases permeability and Ca2+ influx. Carotid sinus afferent fibers, which project to nucleus of the solitary tract (NTS), trigger the excitation of NTS neurons and finally hyperventilation (Figure 1) (Gourine and Funk, 2017; Zera et al., 2019). The NTS is an integrating center that receives information sensed by the CB and stimulates other respiratory centers to trigger the hypoxic ventilatory response (HVR). Alternatively, Torres-Torrelo et al. (2021) proposed a novel CB activation model, which theorizes the existence of a metabolic pathway that is mediated through lactate signaling. Indeed, the authors proposed that lactate is transported into the cells by monocarboxylate transporters 2 and 4, which are rapidly converted to pyruvate with the production of NADH, which correspondingly activates membrane cation channels to produce cell depolarization (Torres-Torrelo et al., 2021). Notably, the increase in NADH production may be facilitated by intracellular acidification and promoted by hypoxemia (Torres-Torrelo et al., 2021). In addition, pyruvate can also increase the production of reactive oxygen species in the mitochondria, which could also contribute to the activation of CB glomus cells (Iturriaga et al., 2021; Torres-Torrelo et al., 2021). Although the CB activation mechanism could be different, the NTS, through glutamatergic synapses toward the RVLM, increases sympathetic discharge to the heart and blood vessels, which is concomitant with an increase in the respiratory drive (Figure 1). Therefore, the CB-mediated control of ventilation and sympathoexcitation is finely coordinated between the peripheral sensors and neuronal nuclei at the brainstem.

In addition, it has been shown that the sensitivity of the central chemoreceptor to hypercapnia is also dependent on CB afferent activity to control ventilation (Paula-Ribeiro and Rocha, 2016). The interaction between both chemoreflex mechanisms is still controversial. However, the activation of peripheral and/or central chemoreceptors is apparently dependent on each other. In support of this notion, Smith et al. (2015) studied this interaction in non-anesthetized dogs by stimulating central chemoreceptors with hypercapnia, through a vascularly isolated and extracorporeally-perfused CBs preparation. They concluded that the relationship between central and peripheral chemoreflex is hyperaddictive or synergistic where stimulation of one chemoreflex increases the response of the other (Smith et al., 2015). However, evidence is limited regarding the adaptations of these mechanisms associated with breath-hold at rest and during exercise. Of note, our group recently showed that swimmer athletes displayed a decrease in CB-mediated respiratory responses to hypoxia compared to a control condition (Arce-Alvarez et al., 2021). Therefore, when considering the evidence, it is possible to hypothesize that these athletes displayed both peripheral (CB sensor) and/or central alterations, which could confer greater resistance to the early termination of maximum voluntary apnea.

### Central Chemoreflex

Central respiratory chemosensitivity refers to a homeostatic reflex by which brainstem circuits regulate breathing in response to changes in CO2, HCO3- or pH of cerebrospinal fluid (Kumar et al., 2015; Goncalves and Mulkey, 2018). Central chemoreceptors are located in different brain areas including the brainstem, ventral medulla, cerebellum, hypothalamus, and midbrain (Nattie and Li, 2012; Gourine and Dale, 2022). Neuronal and glial groups of the retrotrapezoid nucleus (RTN), solitary tract nucleus (NTS), locus coeruleus, fastigial nucleus, or pre-Bötzinger are cell groups that generate the ventilatory reflex adjustments under different insults. Despite the numerous nuclei with chemosensory cells, the RTN is considered a critical nucleus in central chemoreception (Guyenet, 2012) due to this area accounts for ∼90% of the total central chemoreflex drive during exposure to hypercapnia (Kumar et al., 2015). Neurons in this nucleus detect brain extracellular fluid acidification by increasing PaCO2, thus promoting ventilatory adjustments to regulate CO2 excretion (Kumar et al., 2015). Mechanistically, RTN neuron chemosensitivity depends on the expression of proton-sensing G-protein coupled receptor type 4, which is activated by an increase of [H+] in the cerebrospinal fluid; this effect correspondingly produces a closed tandem pore domain in weakly rectifying K+ channels (TWIK), increasing the membrane potential and producing neuron depolarization and finally hyperventilation (Kumar et al., 2015) (Figure 1). Interestingly, Goncalves and Mulkey (2018) showed that RTN neurons possibly also respond to changes in extracellular HCO3- by mechanisms independent of K+ sensing. Particularly, they showed that changes in HCO3-, above or below physiological levels, modify the activity of the chemosensitive neurons of the RTN; however, the mechanistic evidence that this signaling pathway is still limited (Goncalves and Mulkey, 2018). In addition, RTN neurons can control breathing patterns through a glutamatergic projection to the pontomedullary regions (Guyenet, 2012). Indeed, RTN depolarization of glutamatergic neurons produces an activation of the respiratory pattern generator and sympathoexcitatory vasomotor neurons from the rostral ventrolateral medulla, thus generating sympathoexcitation toward tissues and causing a positive chronotropic and inotropic effect of the heart, which increases the tone of blood vessels in accordance with hyperventilation (Moreira et al., 2006) (Figure 1). In addition, glial cells from RTN have also been shown to play an important role in central chemoreception signaling. Current evidence shows that RTN astrocytes cells also have chemosensory functions and respond to changes in CO2 and H+ (Gourine and Dale, 2022). Nevertheless, the mechanism by which astrocytes participate in the respiratory drive induced by hypercapnia is not well described. However, it has been proposed that CO2-induced intracellular acidification of astrocyte cells stimulates Na+ influx mediated by the opening of the Na+/HCO3- NBC cotransporter (Gourine and Dale, 2022). The increase in voltage produces activation of the Na+/Ca2+ exchanger (NCX), producing the influx of Ca2+, which allows the release of ATP by exocytosis from the astrocyte towards extracellular space (Mulkey and Wenker, 2011; Gourine and Dale, 2022). The ATP released by the astrocytes activates purinergic receptors of the RTN chemoreceptor neurons, triggering depolarization of these neurons and stimulating the central pattern generator (CPG) cells through excitatory glutamatergic synapsis producing the ventilatory drive. Therefore, when considering that peripheral and central chemoreflex activation is apparently dependent on each other (Paula-Ribeiro and Rocha, 2016) and swimmer athletes display a reduction in HVR (Arce-Alvarez et al., 2021), it is plausible to hypothesize that water sports athletes exhibit peripheral and/or central chemoreceptor alteration mechanisms, which are able to increase the apnea time by retarding or decreasing the ventilatory drive stimulated by CO2/H+ accumulation (hypercapnia) and/or arterial O2 reduction (hypoxia) induced by a breath hold. Although the evidence studying the ventilatory response to hypercapnia (HCVR) is limited, studies have shown that in elite breath-hold divers the HCVR is blunted with respect to control subjects, which could be an adaptive response to their training regimen and would allow them to maintain prolonged times of sub-aquatic apnea (Grassi et al., 1994).

## Central and Peripheral Interaction: A Key Point in Autonomic RESPONSE to Voluntary Apnea

Apnea involves voluntary (i.e., in aquatic immersion sports) or involuntary (i.e., central and/or obstructive sleep apnea) cessation of ventilation, which decreases PaO_2_ (hypoxemia) and increases PaCO_2_ (hypercapnia) (Sasse et al., 1996). The cessation of ventilation promotes several physiological protection mechanisms against O_2_ deprivation, which involves a decrease in metabolic demands and the redistribution of blood flow to vital organs, such as the brain and heart (Bouten et al., 2020). These autonomic responses are mediated by signaling circuits that are dependent on the central and peripheral chemoreceptors. Thus, peripheral and central chemoreflexes, despite depending synchronously on each other and working under independent mechanisms (St Croix et al., 1996; Barash et al., 2009; Paula-Ribeiro and Rocha, 2016), can both be activated during a maximum breath-hold. Notably, several studies have shown that chemoreflex stimulation promotes an increase in sympathetic activity, thus generating a positive chronotropic response and vasoconstriction to ensure cerebral blood flow (Kara et al., 2003; Keir et al., 2019; Stuckless et al., 2020). However, the possible hypothetical role of chemoreflex control during a voluntary breath hold at rest and during exercise (dynamic apnea) has only been determined under controlled conditions and not in a natural environment for swimmer athletes (Arce-Alvarez et al., 2021; Bruce et al., 2021; Holmström et al., 2021). Interestingly, although the chronotropic response following central and/or peripheral chemoreflex stimulation in aquatic immersion sports is well documented, the results have been controversial. In fact, Trembach and Zabolotskikh (2017) showed that transient CO_2_ inhalation was associated with maximum voluntary apnea in healthy individuals; however, Bain et al. (2017) showed that the central respiratory chemoreflex, through the mechanism of stimulation by hyperoxic rebreathing, was not related to maximum breath-hold duration. Of note, while Trembach and Zabolotskikh (2017) recruited healthy participants, Bain et al. (2017) recruited apnea divers. Therefore, the controversial results could be related to the study population and possibly explained by the heterogeneity with which the chemoreflex was determined. Additionally, other limitations that can explain the controversial results could be related to pharmacological approaches. Indeed, no studies have focused on chemoreflex activity via the use of pharmacological strategies to reduce or increase chemoreflex control in swimmer athletes. Additionally, the aforementioned studies have mostly focused on autonomic and cardiovascular responses, thus excluding other organs that could contribute to the maintenance of oxygen saturation despite not experiencing ventilation. One of these organs could be the spleen, which has been shown to play an important role in the responses and adaptations to hypoxic training; however, studies of this organ in voluntary apnea conditions are extremely limited (Engan et al., 2013; Pernett et al., 2021).

## Spleen-Chemoreflex Relationship in Voluntary Apnea

The spleen is an intraperitoneal organ and is a fundamental part of the reticuloendothelial system, with key implications for the immune response (Baas et al., 1994:; Kapila et al., 2021). In addition to the production and differentiation of immune cells, the spleen is considered a reservoir organ for oxygenated blood, with this organ able to store approximately 215 ml of blood in the normal population and approximately 336 ml of blood in elite divers (Prassopoulos et al., 1997). This reservoir of blood, which is delivered into the systemic circulation, is capable of contributing to maintaining oxygen saturation for a long time period, despite not experiencing pulmonary ventilation (Bakovic et al., 2003; Richardson et al., 2008). Additionally, it can maintain hemoglobin levels during prolonged voluntary apnea or short-term exposure to eupneic normobaric hypoxia (Bakovic et al., 2003; Richardson et al., 2008; Lodin-Sundström and Schagatay, 2010; Pernett et al., 2021). The blood contained in the spleen is released into the systemic circulation via splenic contraction, which is dependent on sympathetic activity (Bakovic et al., 2013). As has been previously observed, decreases in arterial O_2_ and/or increases in CO_2_ pressure produce an increase in sympathetic activity that is mediated by peripheral and central chemoreflexes, respectively, which correspondingly produce spleen contraction. Sympathetic-mediated spleen contraction occurs because the spleen is almost completely innervated by sympathetic nerve fibers, which occupy approximately 98% of the total splenic nerve (Williams et al., 1981). An increase in sympathetic activity, as well as an increase in the release of catecholamines, have been shown to decrease the spleen volume and increase the hematocrit; nevertheless, hypoxia, exercise, and any type of stress can produce contraction of the spleen (Stewart and McKenzie, 2002) (Figure 3). Similarly, Ilardo et al. (2018) showed that aborigines of the Bajau tribe, which is a tribe of extreme immersion hunters, displayed significant adaptation at the spleen level that allows them to maintain long-term immersion times. Indeed, aborigines of the Bajau tribe exhibited an increase in the size of the spleen, which would allow them to release significant amounts of oxygenated blood into the bloodstream, thus likely contributing to severe apnea times (Ilardo et al., 2018). Accordingly, it has been shown that the most successful swimmers (with a greater emptying of the spleen) exhibit a time difference of 15 s of apnea vs the least successful swimmers (Schagatay et al., 2012) (Figure 3). Therefore, the chemoreceptor-sympathetic drive-spleen axis could be a determinant for maintaining oxygen supply to active muscles during swimming exercises; however, the mechanism is not completely clear.

## Chemoreflex Responses and Adaptations in Immersion Water Sports

One of the more important limitations regarding determining the role of chemoreflexes on breath-hold in immersion sports athletes is related to the experimental setting. In fact, there are few studies that have been conducted in aquatic environments, which limits the conclusions. Conversely, most studies have been conducted in controlled laboratory environments under simulated conditions (Arce-Alvarez et al., 2021). As a consequence of the abovementioned conditions, the results related to ventilatory responses and adaptations to hypoxia and hypercapnia have been controversial. Song et al. (1963) showed that the hypercapnic and hypoxic ventilatory responses were not different between divers and control subjects (Song et al., 1963). Conversely, 18 years later, Masuda et al. (1981) conducted a study in the same subjects who participated in the study of Song et al. (1963) and assessed the ventilatory response to hypercapnia and hypoxia. They concluded that these divers (due to adaptive phenomena derived from their activity) demonstrated a blunted response to hypoxia with a normal response to hypercapnia, compared to the control group (Masuda et al., 1981). Interestingly, 1 year later, Masuda et al. (1981) demonstrated opposing results in a different group of divers. Thus, it is possible to theorize that the results could be participant-dependent and not necessarily due to a special characteristic of divers; nevertheless, this phenomenon is still under discussion.

When regarding sports water activities, the results are also controversial. Indeed, Davis et al. (1987) demonstrated a blunted ventilatory response to hypercapnia in underwater hockey players compared to land athletes. Nevertheless, Bjurstrom and Schoene (1987) showed that the hypoxic ventilatory response was blunted, without changes in hypercapnic ventilatory drive in the national synchronized swim team, compared to a control group. Similarly, breath-hold elite divers displayed no significant differences in autonomic, ventilatory, and cardiovascular responses to hypoxia (Breskovic et al., 2010a) and to hypercapnia (Dujic et al., 2008), compared to a control condition. Additionally, after 1 month of endurance intensity training, apnea divers exhibited normal peripheral chemoreflex regulation, compared to an untrained control group (Breskovic et al., 2010b). Accordingly, it is possible to propose that exercise training may not impact the breath-hold and/or chemoreflex response; however, the effect of apnea training on chemoreflex control has not been extensively studied. Therefore, further research needs to investigate this important issue.

The role of chemoreflex control in breath holding is limited and controversial. Additionally, most evidence is associated with long-term apnea sports (breath-hold divers); nevertheless, the evidence regarding intermittent apnea sports is even more limited (Ohkuwa et al., 1980; Arce-Alvarez et al., 2021). Indeed, Ohkuwa et al. (1980) showed that the ventilatory response to hypercapnia was markedly reduced in swimmers, compared to an untrained group (Ohkuwa et al., 1980). Additionally, we found similar results, which demonstrated that young swimmer athletes displayed a robust reduction in the hypoxic chemoreflex response, compared to a control group (Arce-Alvarez et al., 2021).

## Practical Applications

Although there is robust evidence depicting ventilatory chemoreflex and breath-hold duration in water sports, mostly these manuscripts recreate aquatic conditions in an environmental laboratory, which could explain, in part, the controversial findings. In addition, most manuscripts fail to determine whether chemoreflex contributed to apnea duration or if apnea training-dependent chemoreflex control. Of note, performance studies have shown that apnea training could positively impact the sports performance of swimmer athletes. Along with this, sub-aquatic apnea training has been shown to be an effective strategy for improving swimming technique at both maximal and sub-maximal intensity (Lemaître et al., 2009). Indeed, two weeks of dynamic apneic training enhanced apnea-induced diving bradycardia increasing the number of heart rate reserve beats (Mulder et al., 2021). Nevertheless, we showed that during a maximum static apnea effort, under laboratory conditions, the HR decreased less compared to a control condition, which apparently was a sympathetic-mediated effect (Arce-Alvarez et al., 2021). In addition, the combination of pre-competitive warm-up plus apnea exercise was shown to improve 400-m performance during a swimming race (Robertson et al., 2020). Therefore, apparently, apnea training could be a feasible maneuver that is able to promote an improvement of competitive performance. However, until now there is no evidence showing both, the real role of chemoreflex control on apnea duration and their possible effects on sports performance. In fact, it is not possible so far to rule out whether the chemoreflex is just a consequence of freediving training, with no role in performance. Therefore, further manuscripts should address this important question.

## Conclusion

**FIGURE 2:**
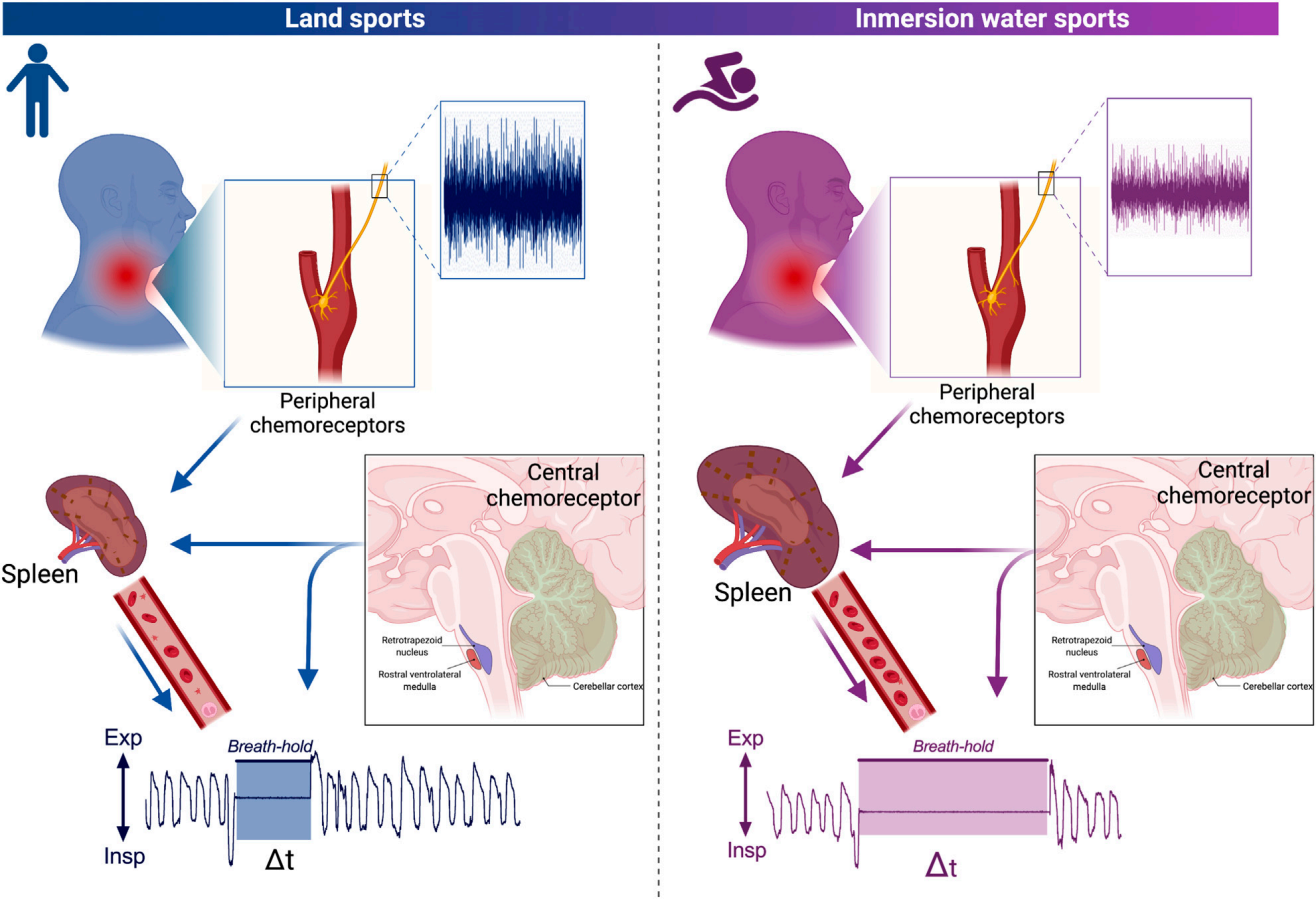
Hypothetical schematic representation model related to the influence of the central and peripheral chemoreflex on a breath hold. Peripheral and central chemoreflex responses of land sports athletes and immersion water sports athletes (left and right, respectively). Carotid body peripheral chemoreceptors send afferences to the nucleus of tractus solitary (NTS) in response to decreases of arterial O_2_ (hypoxia). Accordingly, the NTS project to the central pattern generator (CPG), which is able to produce hyperventilation. In addition, activation of peripheral and central chemoreceptors increases sympathetic activity, producing contraction of the spleen which releases oxygenated blood into the bloodstream. Note that adaptations in immersion athletes involve desensitization of peripheral chemoreceptors which possibly decreases input to NTS and thus to CPG, as well as an increase in size and storage of oxygenated blood in the spleen which allows greater release of blood into the systemic circulation increasing the time of apnea. Nevertheless, apparently central chemoreceptors do not have greater relevance in the breath-hold.

Immersion water sports athletes are characterized by long-term breath holding; however, the possible role of peripheral O_2_ and/or central CO_2_ chemoreflex control is not completely clear. In fact, although chemosensitivity (peripheral and/or central) at rest could be reduced, marked sympathoexcitation during a maximum voluntary breath-hold in young swimmers has been observed. Importantly, the sympathetic drive could activate the spleen, which is a reservoir organ for oxygenated blood, thus contributing to the maintenance of apnea (Figure 2). However, although the chemoreflex could apparently be related to breath holding in swimmer and diver athletes, the evidence is limited. In fact, the mechanism explaining long-term apnea is currently unknown. Therefore, future studies should elucidate whether the explanation to breath hold is related to the sensor (peripheral and/or central chemoreceptors), central command (respiratory and autonomic nuclei at brainstem level), or effectors through the respiratory drive (i.e., respiratory muscles).
